# Angiotensin II-Induced Arterial Thickening, Fibrosis and Stiffening Involves Elevated Arginase Function

**DOI:** 10.1371/journal.pone.0121727

**Published:** 2015-03-25

**Authors:** Anil Bhatta, Lin Yao, Haroldo A. Toque, Alia Shatanawi, Zhimin Xu, Ruth B. Caldwell, R. William Caldwell

**Affiliations:** 1 Department of Pharmacology and Toxicology, Georgia Regents University, Augusta, Georgia, United States of America; 2 Vascular Biology Center, Georgia Regents University, Augusta, Georgia, United States of America; 3 Charlie Norwood VA Medical Center, Augusta, Georgia, United States of America; Albany Medical College, UNITED STATES

## Abstract

**Background:**

Arterial stiffness (AS) is an independent risk factor for cardiovascular morbidity/mortality. Smooth muscle cell (SMC) proliferation and increased collagen synthesis are key features in development of AS. Arginase (ARG), an enzyme implicated in many cardiovascular diseases, can compete with nitric oxide (NO) synthase for their common substrate, L-arginine. Increased arginase can also provide ornithine for synthesis of polyamines via ornithine decarboxylase (ODC) and proline/collagen via ornithine aminotransferase (OAT), leading to vascular cell proliferation and collagen formation, respectively. We hypothesized that elevated arginase activity is involved in Ang II-induced arterial thickening, fibrosis, and stiffness and that limiting its activity can prevent these changes.

**Methods and Results:**

We tested this by studies in mice lacking one copy of the ARG1 gene that were treated with angiotensin II (Ang II, 4 weeks). Studies were also performed in rat aortic Ang II-treated SMC. In WT mice treated with Ang II, we observed aortic stiffening (pulse wave velocity) and aortic and coronary fibrosis and thickening that were associated with increases in ARG1 and ODC expression/activity, proliferating cell nuclear antigen, hydroxyproline levels, and collagen 1 protein expression. ARG1 deletion prevented each of these alterations. Furthermore, exposure of SMC to Ang II (1 μM, 48 hrs) increased ARG1 expression, ARG activity, ODC mRNA and activity, cell proliferation, collagen 1 protein expression and hydroxyproline content. Treatment with ABH prevented these changes.

**Conclusion:**

Arginase 1 is crucially involved in Ang II-induced SMC proliferation and arterial fibrosis and stiffness and represents a promising therapeutic target.

## Introduction

Cardiovascular disease (CVD), a major cause of morbidity and mortality in many parts of the world, is associated with many risk factors such as high blood pressure and cholesterol, diabetes, smoking and stress [1]. Elevated activity of arginase, a urea cycle enzyme, has been implicated in vascular problems such as vascular complications of diabetes, hypertension, aging, coronary artery disease, ischemia reperfusion injury and erectile dysfunction [2–7]. Arginase hydrolyses L-arginine into urea and ornithine and can reduce L-arginine availability for nitric oxide synthase (NOS) [8]. Thus it can reduce NO production, uncouple NOS and increase superoxide production, leading to vascular constriction [5,9]. In addition, upregulation of arginase also can elevate levels of ornithine, substrate for both ornithine decarboxylase (ODC) and ornithine aminotransferase (OAT) [10–12]. Ornithine is catabolized by ODC to produce polyamines, which enhance cellular proliferation. Ornithine also is catabolized via OAT into pyrroline-5-carboxylate (P5C), a precursor for synthesis of proline which promotes collagen formation [13] and perivascular fibrosis. Together, these events can lead to vascular intimal hyperplasia, fibrosis and stiffening.

Increased arterial stiffness has been classified as an independent predictor of cardiovascular mortality in diabetic, coronary artery disease, hypertensive, and stroke patients [14–17]. Arterial stiffness is largely dependent on extracellular matrix (ECM) and vascular collagen levels which are regulated by the activity of OAT and proline formation, and smooth muscle mass regulated by ODC activity and polyamine formation [9,18,19]. Stiffness also can be regulated by smooth muscle tone, which is influenced by circulating and endothelium-derived vasoactive mediators including NO and angiotensin II (Ang II) [20]. Our previous work has shown that diabetes-induced coronary perivascular fibrosis and elevation of carotid artery stiffness in WT mice are reduced in mice lacking one copy of arginase 1 [3]. Another study has shown that an arginase inhibitor S-(2-boronoethyl)-l-cysteine (BEC) prevents loss of arterial compliance in atherosclerotic mice [21]. BEC can exert anti-proliferative effects on VSM by decreasing levels of ornithine, a precursor of polyamines [21]. BEC also can exert anti-fibrotic effects through decreased collagen synthesis via reduced availability of ornithine for OAT to produce hydroxyproline and collagen. Furthermore, aorta medial thickness, wall/lumen ratio, and collagen type I content were found to be much lower in spontaneously hypertensive rats (SHR) treated with an arginase inhibitor (nor-NOHA) compared to untreated SHR [22].

Arginase exists in two isoforms that are encoded by two different genes [23]. Arginase 1 (ARG1), a cytosolic isoform, is located primarily in the liver and assists in the urea cycle. Arginase 2 (ARG2) is mostly mitochondrial and is expressed mainly in extra-hepatic tissues such as kidney and brain [24]. Both ARG1 and ARG2 are expressed in vascular endothelial and smooth muscle cells [8] and their expression is known to be enhanced by inflammatory cytokines and ROS [25–27]. We have previously shown protective effects of pharmacological inhibition and genetic knockdown of arginase against vascular endothelial dysfunction in models of diabetes and hypertension [2–4]. However, the role of arginase in vascular remodeling and arterial stiffness in these pathologies is not well understood. Angiotensin II (Ang II) is known to cause vascular inflammation, ROS production, arginase expression and structural changes not related to elevated blood pressure [28–30]. We hypothesized that elevated arginase activity is involved in Ang II-induced arterial thickening, fibrosis, and stiffness and that limiting its activity can be a therapeutic measure.

## Material & Methods

### Ethical approval

All the animal experiments were approved by the Institutional Animal Care and Use Committee of the Georgia Regents University (animal welfare assurance no. A3307-01). Mice were anesthetized by intraperitoneal injection of a mixture of ketamine (100 mg/kg) and xylazine (10 mg/kg) to allow implantation of minipump in the midscapular region. Animals were housed in a temperature- and light-controlled facility and allowed access to standard chow and water ad libitum. Before harvest of tissues, mice were given a heavy dose of ketamine /xylazine and exsanguinated.

### Animals and Ang II infusion

Experiments were performed using C57BL/6J WT mice and ARG1^+/-^ (heterozygous knockout for ARG1, WT for ARG2) mice. The ARG1^+/−^ mice were developed and provided by Dr. Steven Cederbaum [31]. Complete knockout of ARG1^-/-^ results in death within 2 weeks due to toxic levels of ammonia. Mice were given subcutaneous infusions of either Ang II (1 mg/kg/day) or saline via osmotic minipumps (model 1004; Alzet Co) for 28 days.

### Cell culture and treatments

Rat aortic smooth muscle cells (RASMCs) were purchased from Cell Applications (San Diego, CA) and cultured in rat smooth muscle cell growth medium (Cell Applications) and maintained in a humidified atmosphere at 37°C and 5% CO2. All experiments were performed with cells from passage 4–8.

### Drugs and chemicals

Acetylcholine, phenylephrine, phosphatase cocktail 1 and 2, and protease inhibitor were purchased from Sigma Aldrich (St. Louis, MO, USA). The arginase inhibitor, 2-S-amino-6 boronohexanoic acid (ABH) was a kind gift from Corridor Pharmaceuticals, Inc., Baltimore, MD.

### Vascular stiffness

Pulse wave velocity (PWV), the standard *in vivo* measure for arterial stiffness, was assessed by Doppler ultrasound (VEVO 2100, Visualsonics). Briefly, mice were anesthetized by 1% isofluorane/oxygen inhalation and maintained during the entire procedure. Mice were in the supine position on a heated platform (37°C) and abdominal hair was removed. Aortic pulse waves were assessed at 2 aortic sites; at the aortic arch and the abdominal aorta proximal to iliac bifurcation. PWV calculation was based on the difference in arrival times of a flow wave at two locations along the aorta of known distance. The R-wave of the ECG is a reference point for calculating arrival time of waves. PWV (m/s) was calculated by dividing the distance by the difference between the two arrival times with an average of 3–5 cardiac cycles.

### Blood pressure measurements

Systolic blood pressure was measured non-invasively by tail-cuff method as previously described [4]. Briefly, animals were trained on alternate days over a period of 10 days to get accustomed to the device. Final measurements were performed on day 28. A total of 15 consecutive readings of the SBP were recorded and averaged.

### Western blot

Lysates from cells or aorta homogenates (20 μg protein) were subjected to electrophoresis on 10% SDS-polyacrylamide gels. Proteins were then electroblotted onto PVDF membranes (Millipore, Billerica, MA). The blots were then blocked with 5% bovine serum albumin (BSA Fraction V, Omni*Pur*) in TBST (0.2% Tween 20 in 1 × Tris-buffered saline). Membranes were then incubated with primary antibodies [anti-Arginase 1, 1:10,000 (Kind gift of Dr. Sidney M. Morris, Jr. of the University of Pittsburgh); anti-type 1 collagen, 1:1000, (Santa Cruz Biotechnology, Inc.); anti-ODC1, 1:2000, (AntibodyVerify); anti-β-actin, 1:4000, (Sigma Aldrich) prepared in 5% BSA solution overnight at 4°C, washed (3x TBST), incubated in secondary antibodies conjugated with horseradish peroxidase for 1 hour at room temperature. Signals were detected using chemiluminescence and analyzed using densitometry.

### Collagen staining

Harvested tissues were fixed in 10% buffered formalin (Sigma-Aldrich, St Louis, MO) and subsequently embedded in paraffin. Paraffin-embedded sections (5 μm thick) were de-paraffinized with xylene and rehydrated by immersion in a graded series of ethanol washes. Aortic collagen content was detected by staining sections with Picrosirius red following manufacturer’s protocol (PolyScientific, Bay Shore, NY, USA). Light microscopy was performed using an Axioplan 2 microscope (Carl Zeiss, Jena, Germany) equipped with an Axiocam HR camera and software (Axiovision 4.6.3; Carl Zeiss). Collagen deposition around the coronary vessels was detected by red staining. Area of collagen staining relative to the vessel surface area was quantified using National Institutes of Health ImageJ software. Perivascular fibrosis data are expressed as the collagen-to-vessel surface area ratio.

### Immunohistochemistry

Immunostaining of the aortic rings to identify proliferating cells with proliferating cell nuclear antigen (PCNA) was performed for confirmation of cellular proliferation [32]. Aortic ring sections (5 μm) were fixed with 4% paraformaldehyde, treated with 3.0% H_2_O_2_ for 15 minutes for endogenous peroxidase quenching, and blocked for 30 minutes in 10% normal goat serum. Sections were then washed with PBS (2x2 minutes) and incubated in 1:1000 anti-PCNA (Millipore) for 1 hour at room temperature. Upon washing, the samples were incubated with Alexa Fluor 488–conjugated anti-mouse secondary antibody for 10 minutes. The sections were then counterstained with DAPI (300 nM) for 2 minutes to display cell nuclei. The samples were quantitatively scored by a third party observer who was blinded to the study. PCNA staining was quantified as a percent of PCNA positive cells (Number of PCNA positive nuclei/Total number of nuclei).

### Hydroxyproline assay

Hydroxyproline levels were quantified using a Hydroxyproline Assay Kit (Sigma, MAK008). Briefly, lysates from cells or whole aortae were homogenized with 12M HCL at 120°C for 3 hours. After homogenization, 50 μl of the homogenate was dried for 3 hours at 60°C incubator to which 100 μl of Chloramine-T solution was added. The resulting mixture was added with 100 μl of 4-dimethylamino benzaldehyde (DMAB) reagent and incubated for 90 minutes at 60°C. The samples were finally read at 560 nm. Values were normalized to the protein concentration of the lysate.

### Vascular function

Following deep anesthesia, aorta were rapidly excised and placed in cold Krebs solution [NaCl, 118 mM; NaHCO_3_, 25 mM; glucose, 5.6 mM; KCl, 4.7 mM; KH_2_PO_4_, 1.2 mM; MgSO_4_ 7H_2_O, 1.17 mM and CaCl_2_ 2H_2_O, 2.5 mM]. Perivascular fat was removed and aorta was cut into 2 mm rings. Rings were mounted in myograph chambers (Danish Myo Technology A/S) filled with Krebs solution at 37°C (pH 7.4) under resting tension of 5.0 mN and continuously bubbled with a mixture of 95% O_2_ and 5% CO_2_. Isometric force was recorded using a Power Lab data acquisition system (Software Chart, Version 5, AD Instrument, Colorado Springs, CO, USA). After equilibration for 1 hour, the ability of rings to develop contraction was assessed by adding KCl (80 mM). Cumulative concentration-response curves to acetylcholine (endothelium-dependent vasodilator) or sodium nitroprusside (endothelium-independent vasodilator) were obtained in rings pre-contracted with phenylephrine (1 μM).

### Arginase activity assay

Rat aorta smooth muscle cells frozen in liquid nitrogen were pulverized in lysis buffer (50 mM Tris—HCl, 0.1 mM EDTA and EGTA, pH 7.5) containing protease inhibitors. The resultant lysates were subjected to three freeze thaw cycle, centrifuged at 14,000 rpm for 10 min and the supernatant were collected. Arginase activity was measured by colorimetric determination of urea formed from L-arginine as previously described [33]. Briefly, the supernatant fraction (25 μL) was heated with 25 μl MnCl_2_ (10 mM, 10 min, 56°C) to activate arginase. The mixture was then incubated with 50 μL of 0.5 M L-arginine (pH 9.7) at 37°C for 1 hr. The reaction was stopped by adding acid; the solution was then heated at 100°C with 25 μl α-isonitroso-propiophenone (9% α-ISPF in ethanol) for 45 min. Samples were kept in the dark at room temperature for 10 min, and absorbance was then measured at 540 nm. Enzyme activity was normalized to the amount of protein assessed by Bradford protein assay.

### Nitric oxide measurement

Production of nitric oxide (NO) synthesis was measured using a Sievers 280i NO Analyzer. Media was collected from treated cell cultures and injected in glacial acetic acid containing sodium iodide in the reaction chamber. NO_2_ is quantitatively reduced to NO under these conditions, which was quantified by a chemiluminescence detector after reaction with ozone.

### Measurement of ODC activity/ expression

Expression of ODC mRNA in cells was measured by Real-Time PCR (RT-PCR) using the primers; 5′-CAGCCTGTGCAGAAGTTTGT-3′ and 5′-TGCACACATTCTCCAATGTCCAATCA-3′ (forward primers) and 5′-TACATTGGCAGAATGGGCTA-3′ (reverse primer). Total RNA was purified suing RNA extraction kit from Ambion Technologies. For ODC activity assay, protein (50 μg) from cellular extracts were brought to 100 μl of ODC assay buffer (25 mM Tris/HCl, pH 7.5, 2.5 mM DTT, 0.1 mM EDTA, 0.2 mM pyridoxal phosphate and 33 mM L-ornithine), containing 0.5 μCi of L-[^14^C]ornithine. Mixture was incubated at 37°C in a 15 mL falcon tube with a 3 mm filter paper soaked with saturated sodium hydroxide solution. The liberated [^14^C] CO_2_ was trapped in the soaked filter paper. The reaction was stopped by the addition of 2N hydrochloric acid and the paper was transferred to a vial containing scintillation fluid. Enzyme activity was measured as the amount of [^14^C] CO_2_ formed, using a liquid-scintillation counter [34].

### Cell proliferation assay

Cell proliferation was determined using the Wst-1 assay (Roche Applied Science), which analyzes the number of viable cells by the cleavage of tetrazolium salts added to the culture medium [35]. Briefly, partially confluent cells were subjected to serum starvation for 48 hrs in a 12 well plate. Afterward, the medium was replaced by DMEM (Dulbecco’s Modified Eagle’s Medium, 10% FBS) with treatments. Following treatments, 50 μl of Wst-1 reagent was added to each wells and incubated for 4 hrs at 37°C. Plates were read immediately on a plate reader at 450 nm. Cell counting also was performed using a hemocytometer with trypan blue staining to confirm cell proliferation.

### Statistical analysis

Data are presented as mean+/-SEM. Statistical analysis was performed using Student's *t*-test or analysis of variance (ANOVA) with a Tukey post-test. *P values* < 0.05 were taken as significant. These analyses were performed using GraphPad Prism, version 4.00 (GraphPAD Software Inc., San Diego, CA).

## Results

### Effect of Ang II infusion on vascular endothelial function

**Fig 1:**
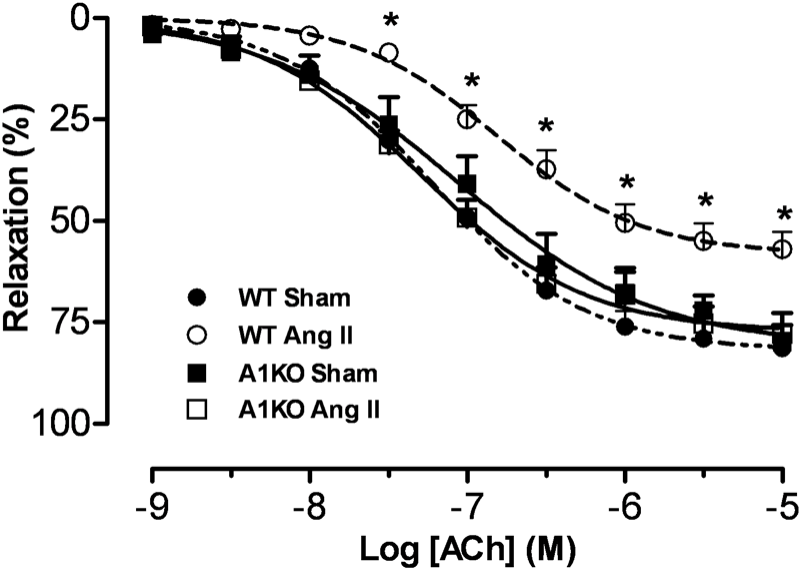
ARG1 deletion prevents Ang II-induced impairment in endothelium dependent vasorelaxation. Aortic rings were pre-constricted with phenylephrine (1 μM). Dashed line/filled circles indicate responses in WT Sham mice, dashed line/open circles indicate responses in Ang II-treated WT mice, solid line/open squares indicate responses in Ang II-treated ARG1^**+/−**^ mice, and solid line/filled squares indicate responses in ARG1^**+/−**^ Sham mice. *n* = 6 in each group; *P< 0.05 vs. other groups.

Our previous study showed that a subcutaneous infusion of Ang II (1 mg/kg/day, 28 days) in mice causes elevated vascular arginase expression and activity, impaired endothelium-dependent vasorelaxation and hypertension that are prevented by co-treatment with the arginase inhibitor BEC (boronoethyl cysteine) or in ARG1^**+/-**^ARG2^**-/-**^ knockout mice [4]. In our present study, a similar vascular dysfunction as evident by reduced relaxation to acetylcholine was observed after 4 weeks of Ang II infusion (1 mg/kg/day) (Fig. 1). In contrast, ARG1^**+/−**^ KO mice (but WT for ARG2) were protected against Ang II-induced vascular endothelial dysfunction (VED). There were no differences among the groups in endothelial-independent vasorelaxation responses to the NO donor sodium nitroprusside (data not shown). These data indicate a role of ARG1 in Ang II-induced VED.

Ang II treatment significantly increased systolic blood pressure (SBP) in WT Ang II (137.2 ± 3.8 mmHg) compared to non Ang II-treated control WT mice (SBP, 110.5 ± 4.5 mmHg). SBP was not significantly raised in Ang II-treated ARG1^+/−^ mice (120.1 ± 5.3 mmHg). SBP in sham ARG1^+/−^ mice (104.9 ± 3.04 mmHg) was not statistically different from control WT mice. These data suggest that the maintenance of vascular endothelial function in ARG^+/−^ mice prevents Ang II-induced rise in SBP.

### Effects of Ang II infusion on aortic stiffness

**Fig 2:**
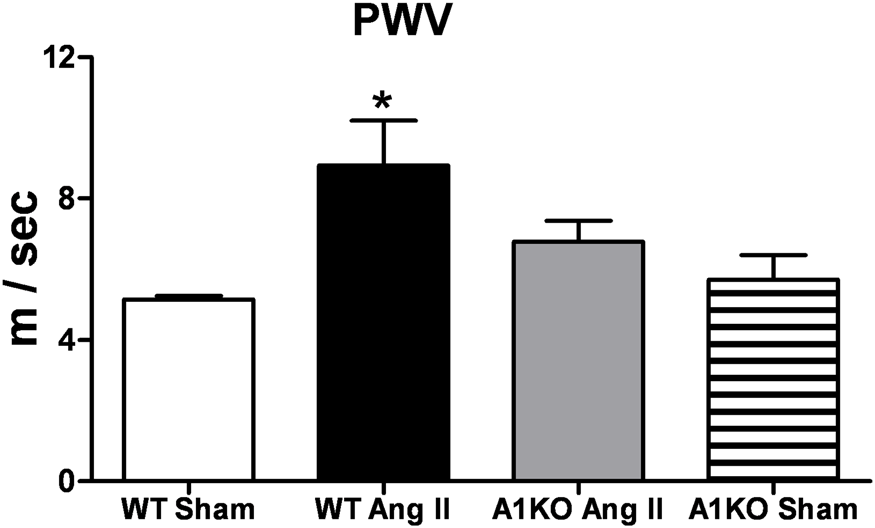
ARG1 deletion prevents Ang II-induced increase in arterial stiffness. Aortic stiffness was measured *in vivo* by pulse wave velocity (PWV) in WT and ARG1^**+/−**^ (A1KO) mice infused subcutaneously for 28 days with angiotensin II (Ang II, 1mg/kg/day) or saline (Sham). Values are expressed as mean+/-SEM, n = 4–6, *P < 0.05 vs. all other groups.

Stiffness of the aorta was assessed *in vivo* by measuring pulse wave velocity (PWV), a proven estimation of elasticity and compliance of large vessels. Enhanced aortic stiffness (loss of compliance), as evident by increased PWV, was significantly increased in Ang II treated WT vs sham mice (Fig. 2). However, aortic stiffness was not elevated in Ang II treated ARG1^+/−^ vs sham mice.

### Effect of Ang II infusion on ARG1 and ODC expression

**Fig 3:**
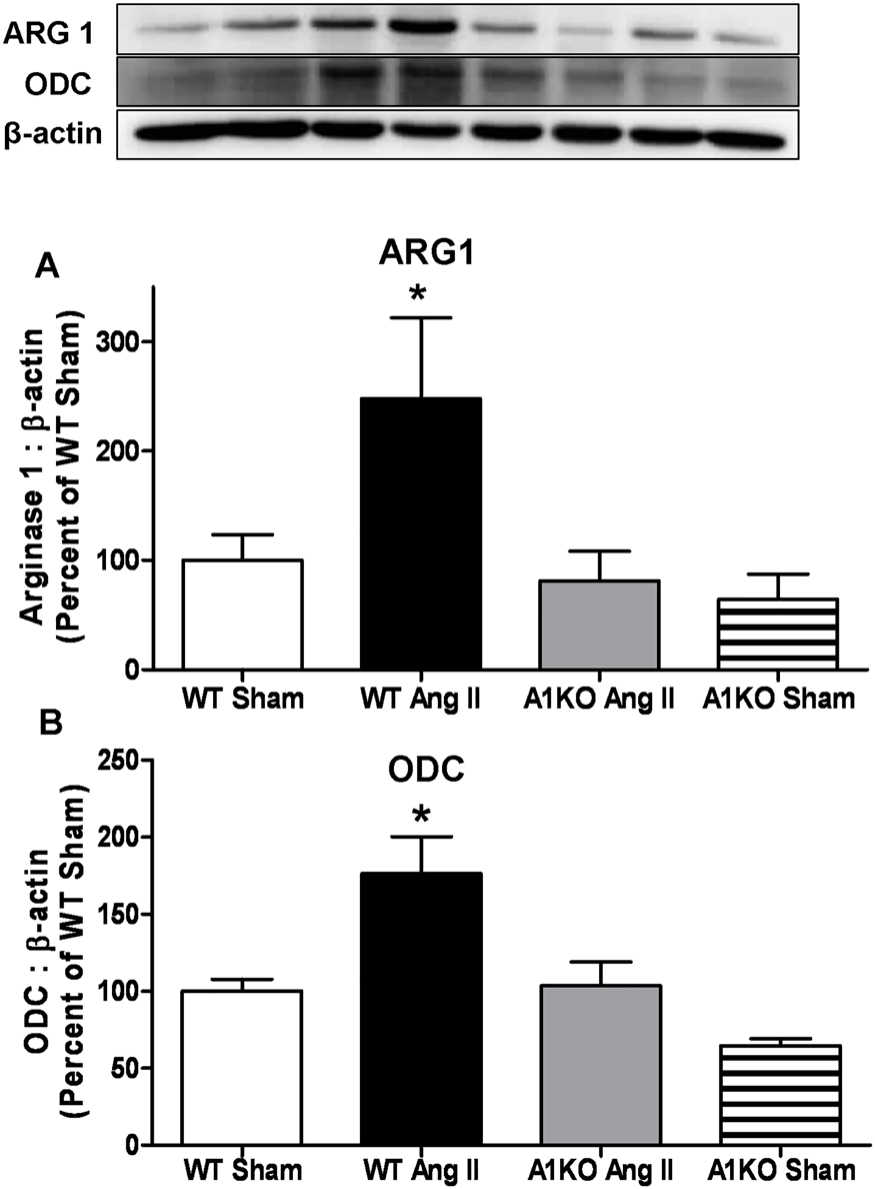
Ang II increases expression of ARG1 and ODC. Levels of **(A)** ARG1 and **(B)** ODC (ornithine decarboxylase) protein expression were determined in WT and ARG1^+/−^ (A1KO) mice infused subcutaneously for 28 days with angiotensin II (Ang II, 1mg/kg/day) or saline (Sham). Western blot results were normalized to β-actin and expressed as percentage of WT Sham. Values are expressed as mean+/-SEM, n = 4–6, *P < 0.05 vs. all other groups.

Aortas excised from WT mice treated with Ang II exhibited increased levels of both ARG1 and ODC protein compared with those of sham WT mice (Fig. 3A and B). Expression of aortic ARG1 and ODC in Ang II-treated ARG1^**+/−**^ mice did not differ from that of the WT or ARG1^**+/−**^ sham mice, indicating that ARG1 mediates Ang II-induced aortic ODC expression. ARG1 expression in ARG1^**+/−**^ mice was not elevated by Ang II treatment. ARG1 and ODC expression tended to be lower in ARG1^**+/−**^ vs WT sham mice.

### Effects of Ang II infusion on aortic thickness and fibrosis

**Fig 4:**
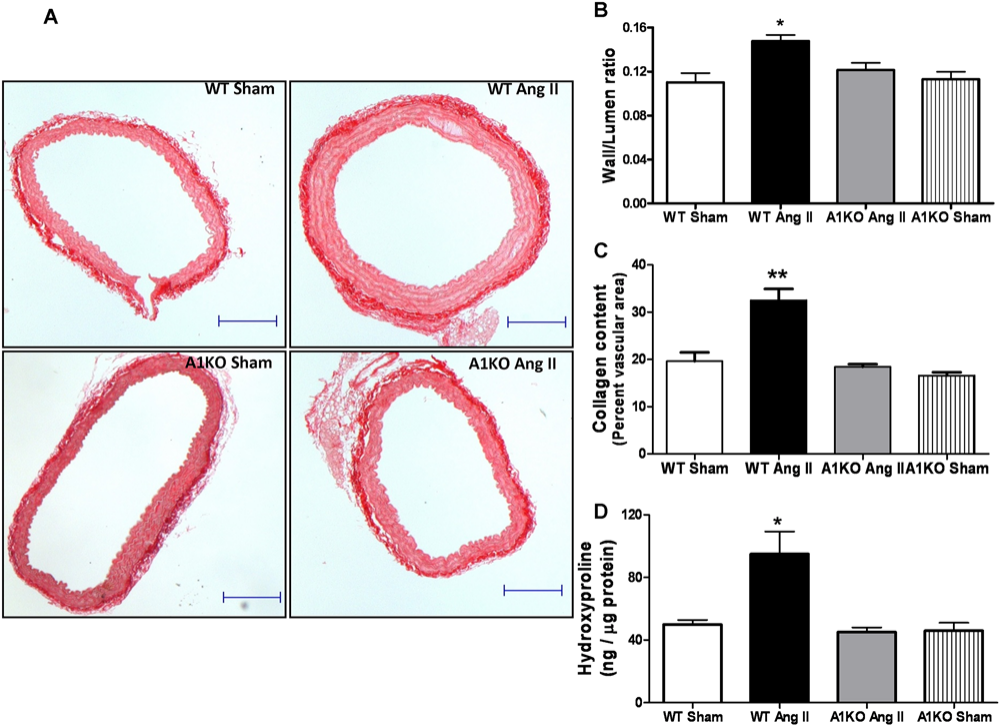
ARG1 deletion prevents increases in aortic wall thickness, fibrosis and hydroxyproline levels in Ang II treated mice. **(A)**, Representative sections of aorta stained with Picrosirius Red for collagen (10x magnification). **(B)** Wall/Lumen ratio and **(C)** Percent collagen per vascular surface area in WT and ARG1^**+/−**^ (A1KO) mice treated with Ang II or saline (Sham). **(D)** Aortic hydroxyproline content. Values are expressed as mean+/-SEM, n = 4–6, *P < 0.05 and **P < 0.01 vs. all other groups. Size bars represent 200 μm.

To determine whether Ang II-induced elevation of aortic stiffness, and increased expression of arginase and ODC were associated with aortic thickness and fibrosis, wall to lumen ratio and collagen as a percentage of vascular area were assessed. Following 4 weeks of Ang II infusion, WT mice had significantly thicker abdominal aorta as evident by increased vessel wall to lumen area ratio (Fig. 4A) and a substantially thicker outer layer of collagen fibers (Fig. 4B). No significant differences in these values were observed between Ang II-treated or sham ARG1^**+/−**^ mice. Ang II treatment also increased hydroxyproline levels in aorta of WT compared with sham mice, but not in aorta of ARG1^**+/−**^ mice (Fig. 4C). This suggests that increased vascular collagen due to Ang II treatment likely occurs via increased arginase activity which results in elevated ornithine/proline pathway, thereby increasing collagen production.

**Fig 5:**
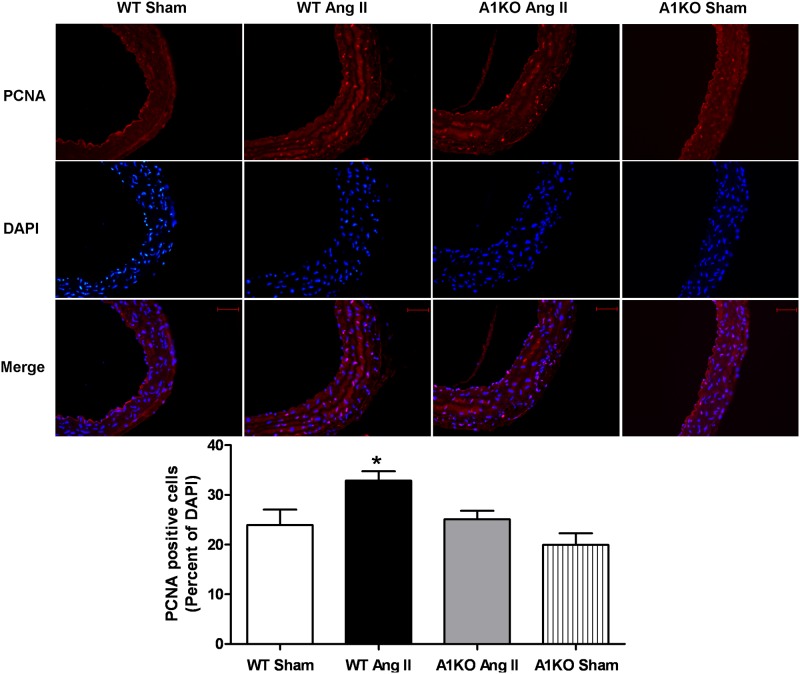
ARG1 deletion prevents Ang II-induced vascular cell proliferation. PCNA was detected following treatments of Ang II (1mg/kg/day, 28 days) or saline (Sham) in WT and ARG1^+/−^ mice. Top, PCNA is stained red and cell nuclei are stained blue (DAPI) (20x magnification). Merged picture shows cells stained with PCNA antibody (red) and DAPI (blue). Bottom, Data presented quantitatively as percentage PCNA positive cells per total nuclei (DAPI stains) (mean+/-SEM, n = 5, 2 sections per mouse). *P< 0.05 vs. other groups. Size bars represent 50 μm.

The effect of Ang II on cell proliferation in the aorta was also examined by staining nuclei in aortic sections with antibody for proliferating cell nuclear antigen (PCNA). Very few PCNA-positive cells were evident in the WT or ARG1^**+/−**^ sham mice. Numerous PCNA-positive cells were observed in the aortic wall of Ang II treated WT mice. PCNA-positive cells were particularly evident in the smooth muscle layer. Ang II treatment produced far fewer aortic PCNA positive cells in ARG1^+/−^ mice than in WT mice (Fig. 5). These results indicate that elevated arginase function mediates Ang II-induced aortic wall thickening, cell proliferation, fibrosis and stiffness.

### Effects of Ang II infusion on coronary artery fibrosis

**Fig 6:**
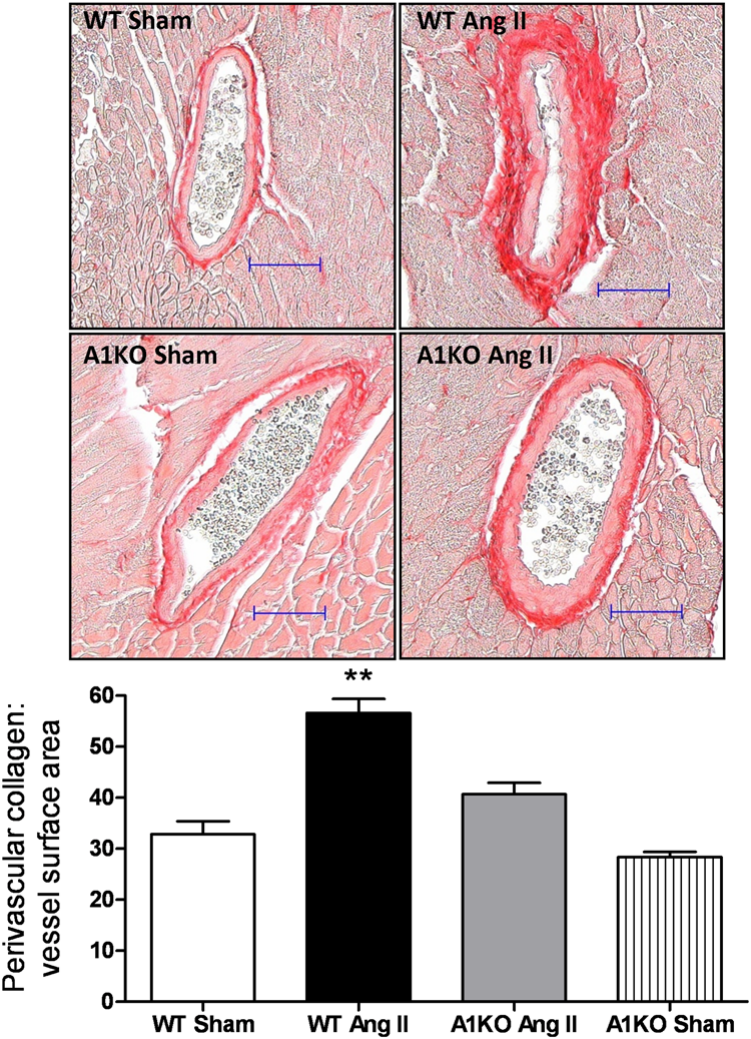
ARG1 deletion prevents Ang II-induced coronary arterial fibrosis. Representative Sirius Red stained heart sections from WT and ARG1^**+/−**^ (A1KO) mice given infusions of Ang II or saline (Sham). Collagen was quantified as its area as a percentage of the vessel surface area. Values are expressed as mean+/-SEM, n = 4–6, *P < 0.05 and **P < 0.01 vs. all other groups. Size bars represent 50 μm.

We next examined perivascular collagen content and fibrosis of the coronary arteries using Picrosirius Red staining. Ang II treated WT mice exhibited enhanced coronary perivascular fibrosis as evidenced by increased levels of perivascular collagen, compared to the WT control (Fig. 6). Perivascular collagen staining appeared slightly higher in Ang II treated ARG1^**+/−**^ mice compared to WT and ARG1^**+/−**^ sham mice, but the values among these groups were not significantly different. These data indicate that Ang II-induced coronary perivascular fibrosis involves elevation of arginase activity, via ARG1.

### Effects of Ang II on arginase activity/expression and NO production in vascular smooth muscle cells

**Fig 7:**
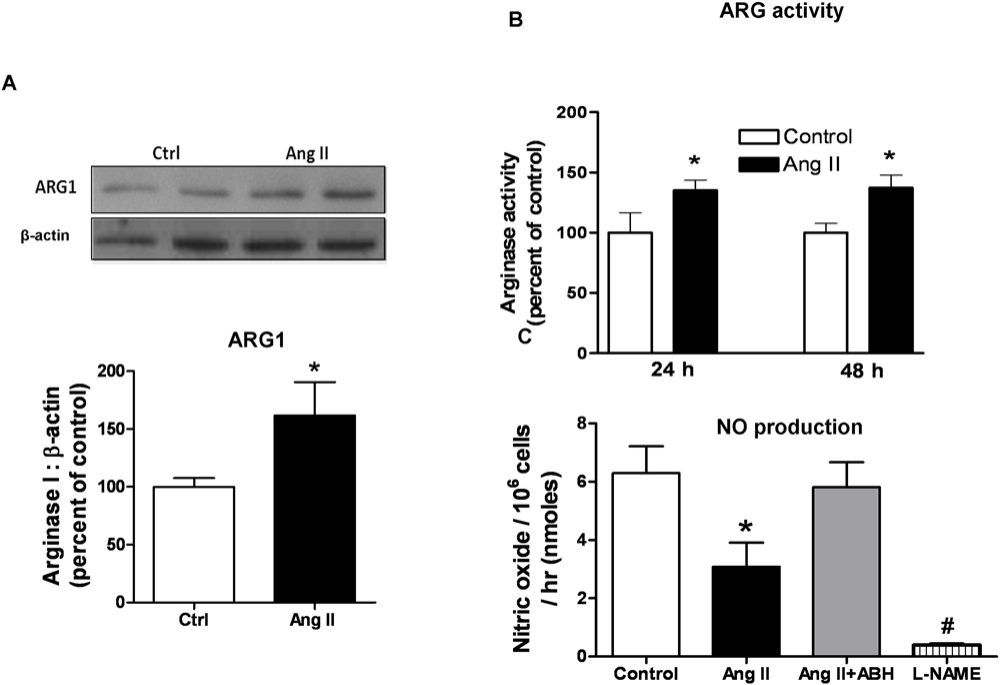
Ang II increases arginase activity/expression and reduces NO production. **(A)** Western blot result for arginase 1 expression, **(B)** arginase activity (colorimetric assay) and **(C)** NO production were measured in RASMCs after control, Ang II (1μM), Ang II+ABH, or L-NAME (1mM) treatment. Arginase activity in control cells was 714.4 ± 36.54 μmol of urea mg^−1^ protein h^−^1. Arginase inhibitor ABH (100 μM) was added 2 hrs prior to Ang II treatment. Values are expressed as mean+/-SEM, n = 4–6, *P < 0.05 vs. all other groups and ^**#**^P < 0.05 vs. all other groups.

Since both our present and prior study [4] indicate that Ang II treatment elevates vascular arginase, we determined the effect of treatment of rat aortic smooth muscle cells (RASMC) with Ang II on arginase activity and expression. Ang II (1.0 μM) treatment for 24 hours caused a 35% increase in arginase activity which was sustained after 48 hours of Ang II exposure (Fig. 7B). ARG1 protein expression was also increased (60%) at 48 hours of treatment (Fig. 7A). Expression of ARG2 did not change with exposure to Ang II (data not shown). This elevated arginase activity was accompanied by concomitant decrease in NO production (36%) at 24 hours (Fig. 7C). Pretreatment (2 hr) of VSMC with the arginase inhibitor ABH (100 μM) prevented this Ang II-induced elevation in arginase activity and reduction in NO production at 24 hr. These data suggest that arginase plays a key role in Ang II-induced reduction of NO production in VSMC. Pre-treatment with the NOS inhibitor L-NAME (1 mM) prevented NO production, indicating NOS as the source of NO. The main isoform of NOS in VSMC is nNOS, but iNOS is induced with inflammation [36,37]. Control studies showed that ABH treatment did not alter expression of ARG1, NO production or cell proliferation (data not shown).

### Effects of Ang II on RASMC ODC activity/expression and proliferation

**Fig 8:**
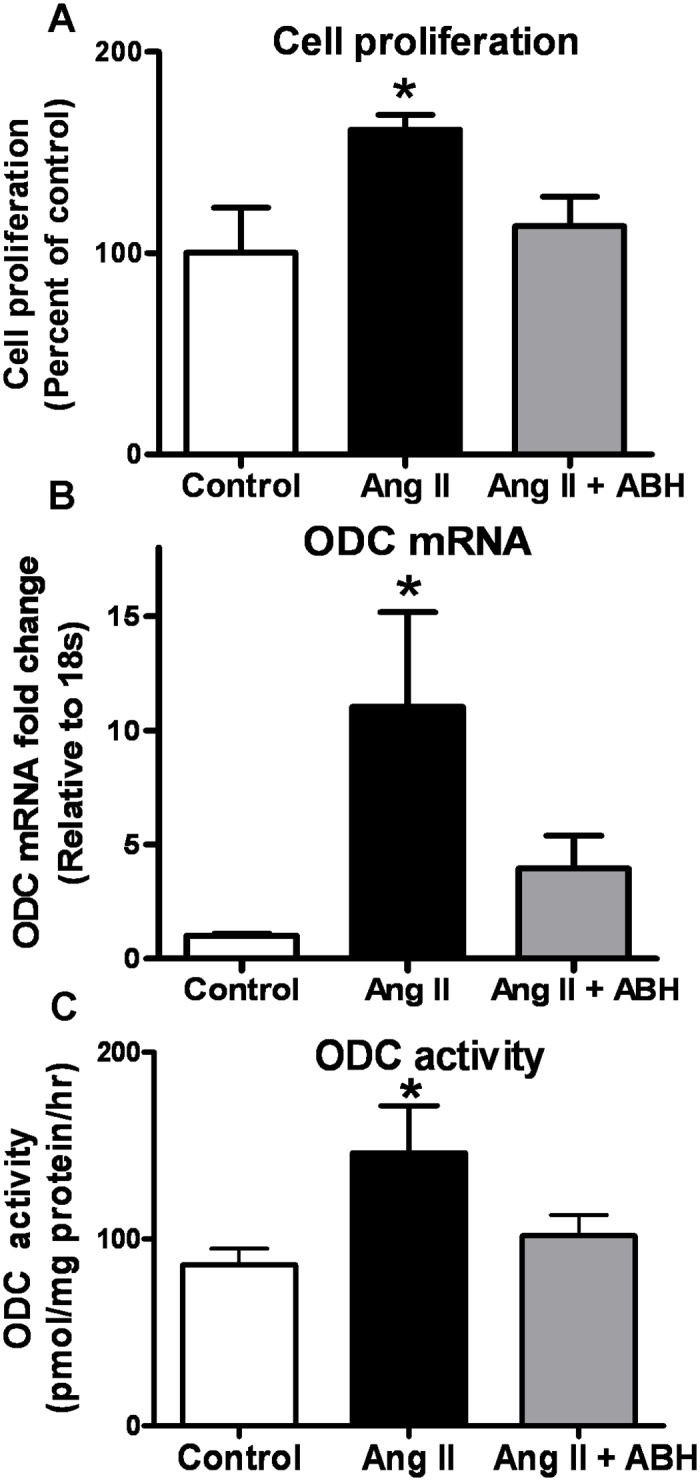
Arginase inhibition attenuates Ang II-induced cell proliferation and ODC activity/expression. **(A)** Cell proliferation, **(B)** ODC mRNA expression (fold change relative to 18s mRNA expression), and **(C)** ODC activity were measured as radiolabelled carbon dioxide production (^14^CO_2_) 48 hrs after Ang II treatment. Arginase inhibitor ABH (100 μM) was added 2 hrs prior to Ang II treatment. Values are expressed as mean+/-SEM, n = 4–6, *P < 0.05 vs. other groups.

It is well known that Ang II can enhance VSMC proliferation [38]. Therefore, we determined whether the Ang II-induced proliferation of VSMC is associated with elevated arginase activity. Exposure of RASMC to Ang II (1 μM, 48 hrs) resulted in an increased cell proliferation (Fig. 8A). This effect was completely blocked by pretreatment of cells with ABH (100 μM), indicating that Ang II-induced proliferation of VSMC is mediated by increased arginase activity. In order to assess the role of ODC in Ang II induced increase in cell proliferation, we measured mRNA levels and activity of ODC in Ang II treated RASMC. Indeed, Ang II treatment induced a 10 fold increase in ODC mRNA and an 80% rise in ODC activity by 48 hrs. Furthermore, pretreatment with ABH blocked the effects of Ang II on both ODC mRNA expression and activity (Fig. 8B and 8C). These data indicate that arginase is involved in the Ang II-induced elevation of ODC expression/activity and cell proliferation.

### Effects of Ang II on RASMC collagen and hydroxyproline levels

**Fig 9:**
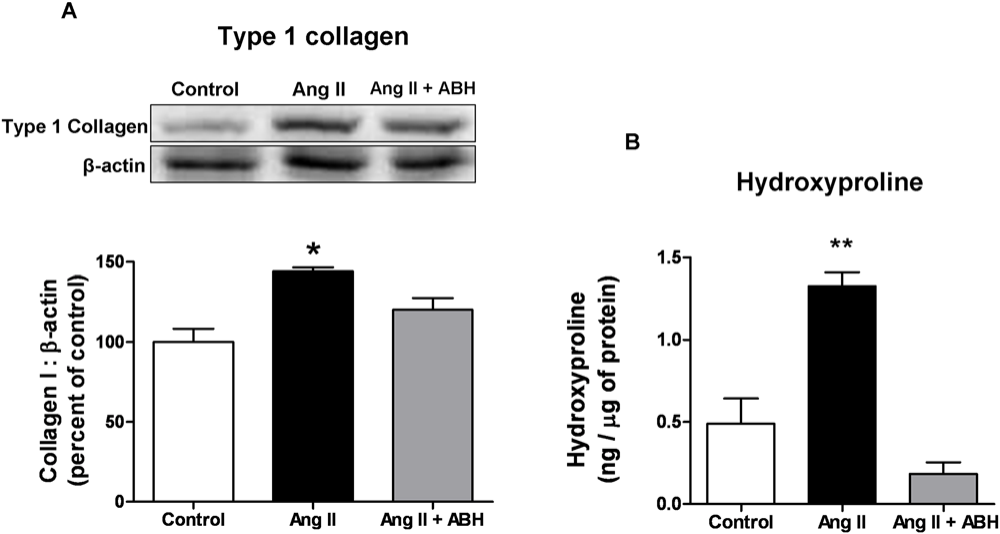
Arginase inhibition blocks Ang II-induced increases in type-1-collagen and hydroxyproline. Collagen and hydroxyproline levels were measured 48 hrs after Ang II or control treatments. **(A)** Top, representative images of type 1 collagen and β-actin expression in western blots. Bottom, levels of collagen normalized to β-actin and expressed as percentage of control. **(B)** Levels of hydroxyproline using an ELISA kit. ABH was added 2 hrs prior to Ang II treatment. Values are expressed as mean+/-SEM, n = 4, *P < 0.05 and **P < 0.01 vs. all other groups.

Ang II also is known to increase levels of several extracellular matrix proteins, including collagen [39]. Since increased arginase activity can provide higher levels of ornithine for the OAT pathway to enhance proline and collagen synthesis, we determined if Ang II-induced arginase activity can also lead to increased collagen expression. Cells treated with Ang II (1 μM, 48 hrs) exhibited a significant elevation of collagen type I protein expression (Fig. 9A). This effect was prevented by pretreatment with ABH (100 μM). Furthermore, hydroxyproline levels were increased in RASMC treated with Ang II after 48 hours compared to non-treated control (Fig. 9B). Hydroxyproline content was not elevated in the Ang II + ABH treated group. These data indicate that arginase is involved in Ang II-induced enhancement of hydroxyproline and collagen levels in VSMC.

## Discussion

In this study, we evaluated the association of arginase 1 with the development of Ang II-induced arterial fibrosis and stiffening in mice. Our main finding is that elevation of arginase activity is critically involved in an Ang II-induced increase in arterial thickness, fibrosis and stiffness. Proliferation of vascular smooth muscle cells (VSMC) plays a pivotal role in the pathogenesis of arterial stiffening and post-angioplastic restenosis [40]. Ang II, a primary effector in the renin-angiotensin system, is a potent stimulus for VSMC proliferation [38,41]. It causes structural changes in arteries involving inflammation and ROS, including increased proliferation of VSMC, accumulation of collagen and fibronectin, and enhanced arterial stiffness [28,39,42]. This is the first study to demonstrate that Ang II promotes VSMC proliferation and collagen synthesis via arginase-dependent polyamine and proline synthetic pathways.

Elevated arginase activity can reduce NO synthesis and contribute to impaired vascular endothelial relaxation [5,43,44]. We have previously shown that Ang II treatment increases arginase activity and expression in endothelial cells and reduces NO production [4]. However, the effects of Ang II on smooth muscle cell arginase have not been examined. Our current study shows that treatment of RASMCs with Ang II increases arginase activity and decreases NO production. Treatment with the arginase inhibitor ABH [2-(S)-amino-6-boronohexanoic acid] prevented the loss of NO production in response to Ang II treatment, indicating involvement of arginase in the suppression of NO levels. Enzymatic sources of NO production in SMCs are neuronal NOS (nNOS) and possibly inducible NOS (iNOS) [36,37]. Although the effect of Ang II on NOS isoforms were not examined in this study, the loss in NO production is due to decreased concentration of NOS substrate L-arginine. In addition to its vasorelaxing actions, NO exerts other beneficial effects, including inhibition of VSMC proliferation. NO can suppress ODC activity in a concentration-dependent manner, via S-nitrosylation of its critical cysteine residues [45]. In addition to reducing NO synthesis by depleting the substrate L-arginine for NOS, enhanced arginase activity also provides more ornithine as substrate for polyamine and hydroxyproline production and can lead to increased cell proliferation and collagen synthesis. Hence, arginase can promote cell proliferation by supplying ornithine for polyamine synthesis through ODC pathway and by reducing NO and its anti-proliferative effect. It has previously been demonstrated that overexpression of arginase 1 increases RASMC proliferation by mechanisms involving increased production of polyamines [18]. We found that Ang II treatment of cultured RAMSCs significantly increased cell proliferation, ODC mRNA and protein expression and ODC activity. These effects, however, were prevented by pretreatment with ABH, indicating that arginase is a mediator of Ang II-induced elevation of ODC expression/activity and cell proliferation.

Vascular collagen synthesis is critical to vascular remodeling and previous studies have reported that Ang II can stimulate SMC collagen production [39,46]. L-Ornithine, a product of arginase, is converted to proline through the ornithine aminotransferase (OAT) pathway. Proline can then be converted into hydroxylproline, a critical component of collagen which is involved in tissue fibrosis. We investigated the relationship between Ang II-induced collagen formation and arginase activity. Our data showed that Ang II increases arginase activity and elevates hydroxyproline production and collagen type 1 protein expression. Furthermore, pretreatment with the arginase inhibitor ABH prevented Ang II-induced arginase activity and substantially inhibited Ang II-induced collagen type 1 protein expression and hydroxyproline formation, indicating that arginase activity is very likely involved in Ang II-induced collagen synthesis.

Increased vascular arginase expression/activity have been previously reported in animal models of Ang II-induced hypertension [4]. Mice lacking one copy of the ARG1 gene and both copies of ARG2 (ARG1^**+/-**^ARG2^**-/-**^) were substantially protected against Ang II-induced impairment of endothelium-dependent vasorelaxation that was seen in WT mice. Furthermore, ARG1^**+/-**^ARG2^**-/-**^ mice and WT mice treated with ABH were partially protected against Ang II induced increase in blood pressure. In support of those findings, we observed protection against Ang II-induced vascular endothelial dysfunction in mice with partial knockdown of ARG1. Moreover, the ARG1 knockdown was protective in mice with both ARG2 genes intact. Our finding suggests a central role for arginase 1 in Ang II-induced endothelial dysfunction.

Hallmarks of negative vascular remodeling are an increase in wall thickness and fibrosis and a reduction in arterial lumen area, associated with reduced arterial compliance [47]. This loss of arterial compliance—stiffening—is now recognized as an important cardiovascular risk factor, independent of blood pressure [48,49]. The medial layer of the vascular wall is composed of SMC and extracellular matrix synthesized by SMC. Increased levels of collagen and proliferation of VSMC can contribute to the development of arterial fibrosis and stiffness. The present results indicate that arginase plays a critical role in collagen synthesis and VSMC proliferation by providing more of the substrate ornithine. Elevated plasma levels of ornithine have been reported in diabetic mice and in diabetic patients that also exhibited increased tissue and plasma arginase activity [50,51]. These results also are in agreement with our previous report in which a partial knockdown of ARG1 in mice was protective against experimental diabetes-induced coronary fibrosis and arterial stiffness [3]. Moreover, pharmacological inhibition of arginase has been shown to block VSMC proliferation and neointima formation in injured rat carotid arteries [52]. In another study, aorta media thickness, wall/lumen ratio, and collagen type I content were found to be much lower in spontaneously hypertensive rats treated with the arginase inhibitor nor-NOHA compared to untreated SHR [22]. These data are supported by our findings of elevated perivascular collagen in the hearts and aorta of WT mice treated with Ang II compared to those from WT control and ARG1 KO mice treated with Ang II. This relation between arginase and collagen is further established by our findings that hydroxyproline levels in WT control and ARG1^**+/-**^mice treated with Ang II were lower than in Ang II treated WT mice.

Collagen content is the net result of a dynamic balance between synthesis and degradation [53]. Reduced collagen synthesis combined with a maintained level of degradation may have deleterious vascular effects as it can lead to disruption of atherosclerotic plaques and release of emboli [54]. Whether arginase inhibition creates a dangerous imbalance in these processes is unclear. Further studies will be required to assess the effects of arginase inhibition on plaque stability in an atherosclerotic animal model.

Clinical studies have demonstrated that cardiovascular disease is associated with stiffening of conduit arteries [47]. Pulse wave velocity (PWV) is a widely accepted non-invasive technique for measuring vascular stiffness *in vivo* [55]. Mechanisms that may account for higher aortic PWV include structural changes in the vessel such as increased vascular collagen content, thickening of the arterial wall and vasoconstrictor tone [52,56]. The present study revealed that Ang II treatment increased PWV in WT mice but not in partial ARG1 KO mice, which exhibited PWV values similar to WT controls. Therefore, our data strongly indicate that ARG1 has a germinal role in Ang II-induced vascular fibrosis and stiffness.

A central question is whether Ang II-induced arterial stiffening is primarily related to elevation of vascular contraction/tone or effects of Ang II that enhance arterial thickness and fibrosis. Many studies have shown a key role of increased Ang II levels in arterial stiffening that involves increased levels of inflammatory cytokines (MCP-1, TNF-α, Il-17), ROS (largely via NADPH oxidase) and TGF-β [28,57–59]. All of these substances are known to increase expression and activity of arginase [7,25–27]. Moreover, reduction of inflammation and ROS can reduce arterial stiffness. Anti-TNF-α therapy in rheumatoid arthritis patients reduces aortic PWV [60]. Blockade of the renal-angiotensin system (ARB or ACEI) in hypertensive patients reduces arterial stiffness (PWV) whereas L-type calcium channel blockers do not, despite producing similar reductions in blood pressure [61,62]. A recent study, examining a treatment that suppresses T-lymphocyte function and IL-17 expression in mice, showed that it prevented Ang II-induced elevation of aortic stiffness/PWV without altering the prominent rise in blood pressure [63]. Thus, we believe that the effects of Ang II on arterial structure—thickness and fibrosis, involving elevated arginase—are chiefly responsible for elevation of arterial stiffness.

The biochemical pathways underlying the effect of Ang II on aortic SMC proliferation, collagen production and arterial stiffening have not been well understood. Our purpose was to determine if elevation of Ang II levels enhance ODC activity/expression, cell proliferation and collagen synthesis and reduce NO production in VSM via an arginase dependent pathway. Additionally, we wished to determine if vascular remodeling in our model of elevated Ang II levels involves upregulation of the ARG, proline and ODC pathways. Our findings provide novel evidence that prolonged Ang II exposure initiates arginase 1-mediated pro-proliferative and pro-collagen synthetic actions in aortic smooth muscle cells leading towards arterial fibrosis and stiffness. Therefore, limiting arginase activity might be a promising pharmacological mean for the prevention and treatment of vascular diseases associated with elevated SMC proliferation and collagen synthesis.
