# Correction: Angiotensin II-Induced Arterial Thickening, Fibrosis and Stiffening Involves Elevated Arginase Function

**DOI:** 10.1371/journal.pone.0127110

**Published:** 2015-04-21

**Authors:** Anil Bhatta, Lin Yao, Haroldo A. Toque, Alia Shatanawi, Zhimin Xu, Ruth B. Caldwell, R. William Caldwell

An affiliation was omitted for the fourth author. Alia Shatanawi is also affiliated with: Department of Pharmacology, Faculty of Medicine, The University of Jordan, Amman, Jordan.
